# Association of Aspiration Pneumonia-Related Factors with the Incidence of Healthcare-Associated Pneumonia in Elderly with Dementia

**DOI:** 10.3390/jcm14176186

**Published:** 2025-09-02

**Authors:** Takahide Miyamoto, Kanae Karita, Koichi Kozaki, Takae Ebihara

**Affiliations:** 1 Department of Geriatric Medicine, Kyorin University School of Medicine, Tokyo 181-8611, Japan; 2 Department of Hygiene and Public Health, Kyorin University School of Medicine, Tokyo 181-8611, Japan; kanae@ks.kyorin-u.ac.jp; 3 Department of General Medicine, Tokyo Jikei Medical University Kashiwa Hospital, Chiba 277-8567, Japan

**Keywords:** swallowing reflex, cough reflex sensitivity, healthcare-associated pneumonia (HCAP), Alzheimer’s type dementia, vascular type dementia

## Abstract

**Background/Objectives**: The predominant etiology of healthcare-associated pneumonia (HCAP) that frequently manifests in elderly with advanced dementia is aspiration pneumonia in which the deteriorated upper respiratory protective reflexes are significant responsible triggers. However, the association of HCAP with cerebral degeneration has not been investigated. Therefore, a cross-sectional and retrospective cohort study was conducted to elucidate the association of aspiration pneumonia-related factors with HCAP in elderly with dementia. **Methods:** Of the 154 participants (87.9 years), 30 of Alzheimer’s type dementia (AD) or 124 of vascular dementia (VaD) were assigned to the pneumonia group or the control group. Participant’s characteristics, including cognition, clinical pattern and stage of dementia, physical and eating abilities, latency of the swallowing reflex (LTSR), threshold of CRS, and tongue moisture (TOM), were evaluated. **Result:** The progression of dementia and the decline in LTSR, CRS, and TOM were synchronized (*p* < 0.05). Participants in the pneumonia group who were male, with eating difficulties, prolonged LTSR, lacunar infarction, or a smoking history, were significantly observed. The multiple logistic analysis indicated that the LTSR was a significant independent factor for developing HCAP (*p* = 0.01). Furthermore, as the possessed number of aspiration pneumonia-related factors increased, the odds ratio for HCAP became significantly higher (*p* < 0.001). Blunted CRS, male gender, and lacunar infarctions were evident in VaD participants but not in AD participants. Finally, the incidence of HCAP in VaD was 2.11 times higher than that in AD (*p* = 0.005). **Conclusions:** The higher incidence of HCAP in VaD than AD may be due to different underlying pathophysiological mechanisms between them.

## 1. Introduction

The preponderance of healthcare-associated pneumonia (HCAP) among disabled elderly individuals has been attributed to aspiration pneumonia (AP), provoked by micro- or macro-aspiration [1].

Approximately half of institutional elderly with dementia experiences HCAP incidence, and approximately 80% of those cases involve eating disorders [2]. Given the significant increase in dementia cases in Japan, which reached 4.7 million in 2025 [3], it is imperative to elucidate the underlying mechanisms that precipitate pneumonia in dementia. Additionally, providing information on the development and prognosis of pneumonia in the context of dementia, as well as preventive measures, is expected to be greatly benefit patients and their families.

According to previous studies, the deteriorated swallowing reflex and cough reflex sensitivity have been identified as significant responsible factors in the development of AP [4]. The input of sensory information via the axonal reflex on the neural ending of the glossopharyngeal and the vagal nerves is afferently received by the central pattern generator located in the medulla oblongata, where it modulates adaptation to the size of the bolus, generates the swallowing rhythm, and relays sensory inputs to the swallowing-related cortical region and motor outputs to their muscles [4].

Furthermore, cerebral infarctions in the basal ganglia have been shown to increase the risk of AP by vulnerable dopamine neurotransmission, SP production, and weakening of the upper airway protective reflex. In addition, xerostomia [4,5], impaired physical function [4], being of male sex [1], smoking history [6], eating difficulty [4], and comorbidities, such as cerebral lacunar infarction [4], chronic obstructive pulmonary disease (COPD) [7], and postoperative conditions of the upper gastrointestinal tract [8], have been identified as other contributing factors of it. Medications that are susceptible to developing or preventing AP have been reported as AP-related factors [9,10].

Heretofore, the comprehensive relationship among AP-related factors, cerebral degeneration, and HCAP has been insufficiently investigated. Therefore, we conducted a cross-sectional and retrospective cohort study to determine the association of AP-related factors with HCAP in elderly individuals with dementia.

## 2. Method

### 2.1. Study Design and Subjects

**Figure 1:**
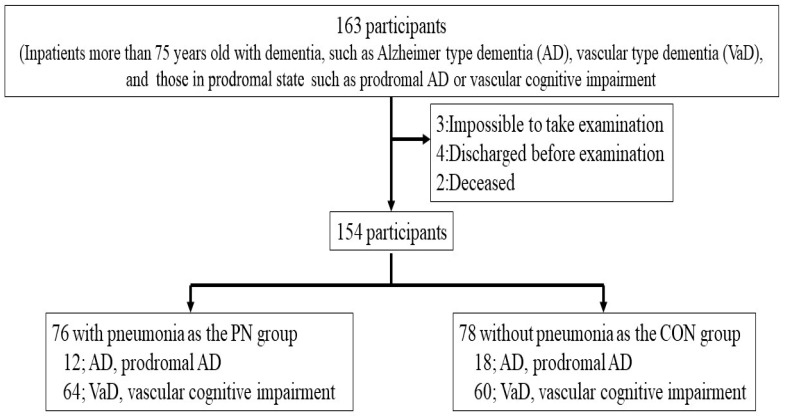
Study design. AD; Alzheimer’s type dementia, VaD; vascular dementia, prodromal AD; prodromal Alzheimer’s type dementia.

A cross-sectional, retrospective cohort study in patients with or without HCAP admitted to our geriatric ward at Kyorin University Hospital was conducted between August 2016 and April 2019 (Figure 1). Patients were excluded if they had community-acquired, hospital-acquired, and ventilator-associated pneumonia, immunosuppression (patients taking steroids for ≥2 weeks, seropositive for human immunodeficiency virus, having received a solid-organ or bone-marrow transplant, on cytotoxic therapy, or with acquired immunodeficiency syndrome), and active tuberculosis.

Inpatients more than 75 years old with dementia, such as Alzheimer’s type dementia (AD), vascular type dementia (VaD), and prodromal AD or vascular cognitive impairment, participated in the study (Figure 1). Out of 163 eligible participants, 9 participants were excluded because they refused to undergo the examination, were discharged from the hospital, or died during the course of the study. Finally, 154 participants with AD (30 participants) or VaD (124 participants) were allocated in the HCAP (PN; 76 participants) or the control group (CON; 78 participants).

The study was approved by our institution’s ethics committee; informed consent was obtained from all participants, or their family members when patients were unable to provide consent, or were diagnosed with dementia using the Mini-Mental Score Examination (MMSE) (approval numbers 684, 701 and 807).

The primary endpoints of the study were the incidence of HCAP among the clinical stages of dementia subtypes. The secondary endpoint was the extraction of AP-related factors that contribute to the development of HCAP.

### 2.2. Diagnosis of Pneumonia and Dementia

Pneumonia was diagnosed by pyrexia, sputum, cough, inflammatory reactions representing increase in the number of leucocytes and C-reactive protein value, and infiltrate shadows with an air bronchogram in the chest X-ray.

Referring to the 2005 American Thoracic Society and Infectious Diseases Society of America guidelines [11], HCAP in the study was defined as elderly individuals who live in nursing homes, were discharge from hospital within 90 days, and needed continuous long-term home care.

AD was diagnosed by the definition following the diagnostic and statistical manual of mental disorders fifth edition (DSM-5) [12]. Morphological diagnosis was executed using computed tomography (CT) or magnetic resonance imaging (MRI) referring voxel-based specific regional analysis system for AD [13]. Additionally, to avoid the effect of cerebral vascular lesions, they were confirmed to not have any visually identifiable cerebral vascular lesions on their brain MRI scans. Furthermore, a decrease in cerebral blood flow in the posterior cingulate and the angular gyrus were also considered an important diagnostic criterion [14]. VaD was also diagnosed by the DSM-5 definition and the report of the NINDS-AIREN International Workshop [15], referring to cerebrovascular disorders such as lacunar infarction and multiple small infarcts of cerebral white matter around the cerebral ventricles on brain images.

### 2.3. Evaluation of Dysphagic Risk in Aspiration Pneumonia-Related Factors

The swallowing reflex of participants was provoked in the supine position. Latency of the swallowing reflex (LTSR) was evaluated as time from the nasal-catheter injection of 1 mL of the distilled water into the pharynx through a nasal tube (ATOM, 4Fr.), observing a visual characteristic movement. Normal values were reported to be less than 3 s [4].

Individual cough reflex sensitivity (CRS) was evaluated as the maximum concentration of citric acid monohydrate-dissolved saline at which patients coughed more than five times when inhaling vaporized solution from 0.7 to 360 mg/mL using an ultrasonic nebulizer (NE-U780^®^, Omron, Japan) for 1 min. A threshold concentration more than 1.35 log mg/mL is recognized as a risk of the development of AP [4].

Tongue oral moisture (TOM) [5] was measured using an oral moisturizer (Mucus^®^, Life Co., Ltd., Saitama, Japan), which was attached 10 mm from the tip of the tongue and recorded the median value of 3 measurements. Evaluation of those AP-related factors was conducted in the morning after recovery from illness, defined as vital signs within the normal range and an afebrile condition.

### 2.4. Other Factors Affecting the Incidence of Aspiration Pneumonia

#### 2.4.1. Comprehensive Geriatric Assessment

The MMSE [16] and the Functional Assessment Staging (FAST) [17], as the cognitive and clinical stage of dementia, the Barthel Index [18], as activities of daily living (ADL), and the Food Intake Level scale (FILS) [19], as individual eating ability, were evaluated. Individual FAST, Barthel Index, and FILS levels before admission to the hospital were evaluated through interviews with their families. The MMSE was scored after recovery from their illness, defined as having normal vital signs and being afebrile.

#### 2.4.2. Aspiration Pneumonia-Related Factors for Lifestyle Habits, Comorbidities, and Medications

The following variables were also included in the study: smoking history, the presence of comorbidities, such as COPD, and postoperative states of the gastrointestinal tract. Furthermore, medications administered prior to admission were examined. Specifically, the investigation encompassed not only antipsychotics [9] and anti-cholinergics [10], which have been demonstrated to be susceptible to the development of AP, but also angiotensin-converting enzyme inhibitor (ACE-I) [4], dopamine-releasing hormone agents [4], and phosphodiesterase-III inhibitors [4], which have the potential to prevent AP.

#### 2.4.3. Cerebral Infarction

Referring to previous reports [4,20,21,22], the number of cerebral infarctions (hemi/bilateral) in the basal ganglia (the caudate nucleus, the putamen/the globus pallidus, the substantia nigra), the frontal orbital gyrus, the insular gyrus, the cingulate gyrus, the primary somatosensory gyrus/the motor gyrus/the supplementary motor gyrus, the internal capsule, the thalamus, the temporal pole gyrus, and the parieto-occipital associated gyrus in CT or MRI, were investigated.

### 2.5. Statistics

We analyzed categorical variables using the chi-square and Fisher’s exact tests. Continuous variables with normal and homoscedastic distributions were analyzed using a *t*-test for two groups and a Kruskal–Wallis test for three groups. Welch’s test was also performed on continuous variables with unequal dispersion. Continuous variables with a non-normal distribution were analyzed using a Mann–Whitney U test. Variables with a non-normal distribution were compared using the Kruskal–Wallis test. The incidence of pneumonia in persons with AD and VaD, extracted at a ratio of 1:1 after adjusting for sex and MMSE, was analyzed using the chi-square test. Multiple logistic analyses were performed to investigate AP-related factors after adjusting for confounding factors. The level of statistical significance was set at *p* < 0.05. Data were analyzed using SPSS v22.0 (IBM Corp., Chicago, IL, USA).

## 3. Result

### 3.1. Patient’s Characteristics

A comparative analysis of the characteristics of 154 participants (74 male participants [48.1%], mean age: 87.9 ± 5.1 years) between the PN and CON groups revealed significant differences in the ratio of sex, the number of participants with a smoking history, and the FILS level. A substantial discrepancy was identified between the two groups concerning smoking history in AD participants, as well as in male and FILS levels in VaD participants (Table 1).

### 3.2. HCAP by Dementia Subtype and Stage

**Figure 2:**
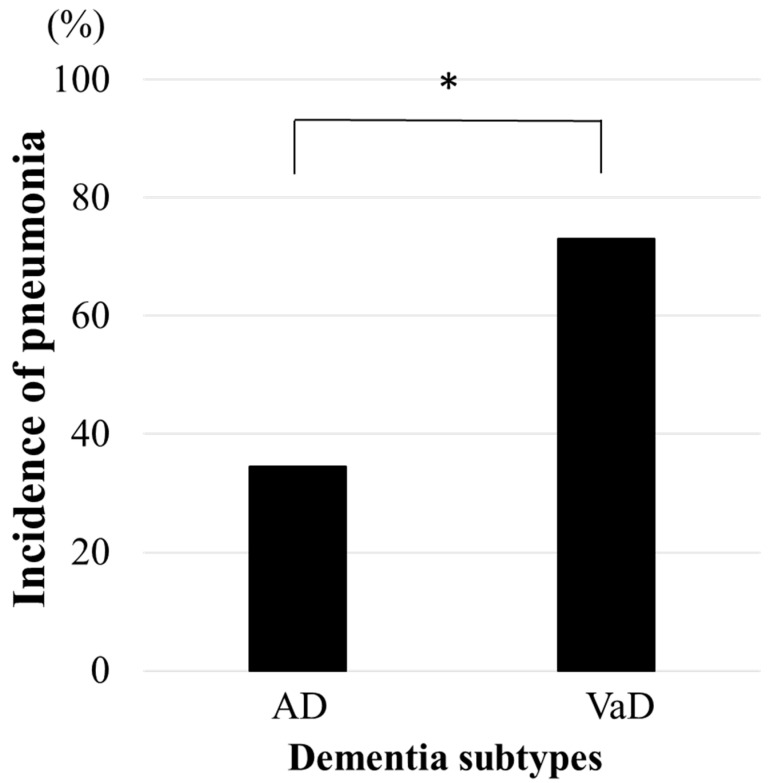
Incidence of pneumonia in the elderly with dementia. AD; Alzheimer’s type dementia, VaD; vascular dementia. Comparison of incidence of pneumonia with sex and MMSE-matched patients. Chi-square test; * *p* < 0.05.

Incidence of pneumonia in the study population was not significantly different between the AD group (40.0%) and the VaD group (51.6%). However, in the comparison of both groups who were extracted at a ratio of 1:1 from the AD and VaD participants after adjusting for sex and MMSE, it was 2.11 times higher in the VaD participants than in the AD participants (*p* = 0.005) (Figure 2). On the other hand, there were no significant inter- and intra- differences in pneumonia incidence among groups of AD and VaD, who were classified into three stages based on MMSE scores: mild cognitive impairment (MCI) with scores ranging from 27 to 23, early to moderate dementia with scores ranging from 22 to 15, and severe dementia with scores ranging from 14 to 0. Specifically, the rates were 60.4% in the MCI stage, 42.6% in the early to moderate dementia stage, and 46.2% in the severe dementia stage (AD: 42.9%, 30.0%, 46.2%; VaD: 63.4%, 45.5%, 46.2%, respectively).

### 3.3. Association Between HCAP and Aspiration Pneumonia-Related Factors by Dementia Subtypes

The LTSR in the PN group showed significantly longer results than those in the CON group (*p* = 0.01). Particularly, such results were more pronounced in the VaD participants (*p* = 0.008, Table 2). Also, a tendency for elevated CRS thresholds in the PN group, especially among VaD participants, was observed (*p* = 0.097, 0.084, respectively), whereas a notable distinction emerged in the LTSR and the CRS between both groups of AD participants.

Additionally, no significant difference in the TOM among all participants, including AD and VaD participants, was observed (Table 2).

A logistic analysis indicated that the LTSR was a significant AP-related factor for developing HCAP in all participants and in the VaD participants (all participants: odds ratio (OR) 3.98, 95% confidence interval (CI) 1.38–11.50, *p* = 0.011, VaD participants: OR 4.71, 95% CI 1.35–16.49, *p* = 0.015). The CRS and the TOM demonstrated no association with the development of HCAP among all participants or VaD participants (Table 2).

### 3.4. The Number of Aspiration Pneumonia-Related Factors in HCAP by Dementia Subtype

The dichotomy analysis indicated that a LTSR of 0.46 log sec. or more and a CRS of 1.35 log mg/mL or more are associated with a higher proportion of HCAP than those with less than the following values in all patients: the LTSR of 0.46 log sec. or more vs. less; 60.8 vs. 43.7 (%), *p* = 0.046, and the CRS of 1.35 log mg/mL or more vs. less; 60.0 vs. 43.4 (%), *p* = 0.049, respectively.

**Figure 3:**
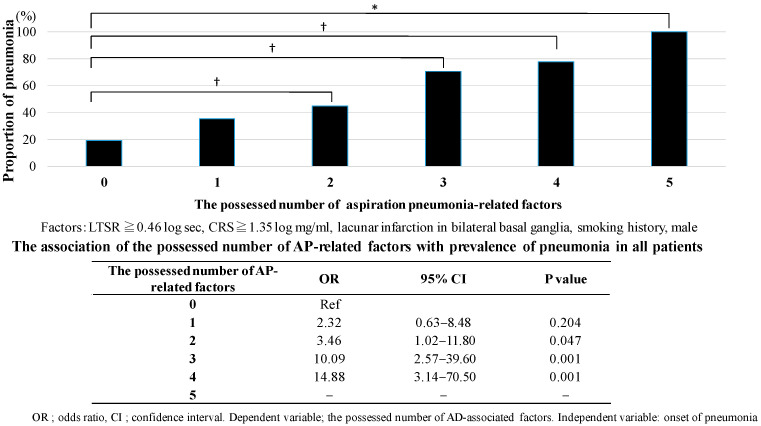
Association of the possessed number of aspiration pneumonia-related factors with incidence of pneumonia in elderly patients with dementia. A latency of swallowing reflex more than 0.46 log sec, a threshold of cough reflex sensitivity more than 1.35 log mg/mL, bilateral lacunar infarction, smoking history, and being male were counted as 1 point and classified into 6 groups (0–5) by the number of possessions of these factors above. Chi-square test, * *p* < 0.05. Logistic analysis, ^†^ *p* < 0.05.

The analysis revealed that participants with both factors exhibiting a LTSR of 0.46 log sec or higher and a CRS of 1.35 log mg/mL or higher, demonstrated a higher incidence of HCAP compared to patients without either factor (the number of factors possessed of 0 vs. 2; OR 4.38, 95% CI 1.58–12.15, *p* = 0.005). Finally, the incidence of HCAP demonstrated a statistically significant association with the number of AP-related factors that were observed, with a LTSR of 0.46 log sec or higher and a CRS of 1.35 log mg/mL or higher, bilateral lacunar infarctions, smoking history, and male sex (*p* < 0.001). As the number of factors increases, the incidence of HCAP concomitantly rises (Figure 3, bottom Table).

Additionally, three factors including blunted CRS, lacunar infarctions, and male sex were not observed in AD but in VaD. As the number of factors increased, the incidence of HCAP increased concomitantly (OR 3.48, *p* = 0.130, 22.50, *p* < 0.001, 19.50, *p* = 0.001).

### 3.5. Relationship Between Aspiration Pneumonia-Related Factors and Cognitive Function by Dementia Subtype

**Figure 4:**
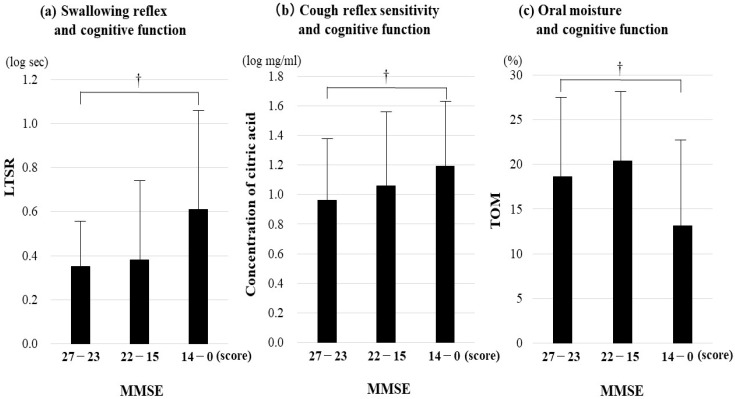
Association of aspiration pneumonia-related factors with cognitive function in the elderly with dementia. (**a**) Swallowing reflex and cognitive function, (**b**) cough reflex sensitivity and cognitive function, (**c**) oral moisture and cognitive function. The tertile categorization across each clinical dementia stages with the subsequent definitions using MMSE is as follows: 27 to 23 points, 22 to 15 points, and 14 and 0 points are designated as the mild cognitive impairment (MCI) stage, the early–moderate dementia stage, and the advanced dementia stage, respectively. Aspiration pneumonia-related factors were compared for each class by Kruskal–Wallis analysis. A post hoc comparison (Bonferroni) for each class. ^†^ *p* < 0.05.

A tertile cognition-based analysis in all participants showed that the LTSR was more prolonged, the CRS was higher, and TOM was lower in the advanced dementia stage than in the MCI stage (post hoc analysis, *p* = 0.015, 0.033, 0.002, respectively) (Figure 4). In AD participants, the LTSR in the advanced dementia stage showed more prolonged results than those in the early–mild dementia stage (post hoc analysis, *p* = 0.023) whereas the CRS and the TOM did not differ significantly among them.

In VaD participants in the advanced dementia stage, a longer LTSR and lower TOM were observed (post hoc analysis, *p* = 0.009, 0.004, respectively).

### 3.6. Association of HCAP with Cerebral Infarction

All participants and those with VaD in the PN group demonstrated a higher tendency for lacunar infarction in the cerebral basal ganglia, particularly on the bilateral side, in contrast to those in the CON group (all; OR 2.74, 95% CI 1.57–4.78, *p* < 0.001, VaD; OR 3.14, 95% CI 1.54–6.42, *p* = 0.002, respectively). This is especially because the proportion of lacunar infarction in the putamen and the globus pallidus in the PN group was greater than those in the CON group (OR 2.74, 95% CI 1.57–4.78, *p* < 0.001). Conversely, no association was observed between the incidence of HCAP and lacunar infarction in AD participants. Also, the cortical infarction in regions such as the superior frontal gyrus, the insular gyrus, the cingulate gyrus, the primary somatosensory/motor/supplementary motor gyrus, the internal capsule, the thalamus, the temporal pole gyrus, and the parieto-occipital association gyrus, was not associated with the incidence of HCAP among all participants, those with VaD, and those with AD.

## 4. Discussion

Our study showed that the incidence of HCAP was 2.11 times higher in the elderly with VaD than in those with AD, and no significant difference among clinical dementia stages among all participants, including those with AD and VaD. A deteriorated swallowing reflex was a key AP-related factor in VaD participants. As the possessed number of AP-related factors increased, so did the OR of developing HCAP. These factors included a deteriorated swallowing reflex, blunted CRS, bilateral lacunar infarction, smoking history, and being of male sex. No AP-related factors in HCAP were identified in AD participants.

### 4.1. Association Between HCAP and the Aspiration Pneumonia-Related Factor by Dementia Subtypes

The study firstly showed that the deteriorated swallowing reflex was an important responsible factor for developing HCAP in elderly individuals with dementia, particularly with VaD but not AD. Similarly to a previous study, another sub-analysis rendered reproducibility that the LTSR in patients with bilateral lacunar infarction was significantly longer than those without such infarctions in all participants (*p* = 0.03). However, the significant association of HCAP with infarctions in the putamen and globus pallidus of the basal ganglia in the VaD participants (*p* = 0.04), but not AD (sub-analysis, shown in RESULT), is highly novel, in sight of interpreting the mechanism of prolonged LTSR suggesting the presence of micro-aspiration even in MCI stage.

As already established, substance P is a critical neurotransmitter synthesized by the dopaminergic and substance P-containing neuron in the putamen and the globus pallidus, which elicits upper respiratory defensive reflexes [4]. Therefore, the presence of bilateral cerebral infarctions in the putamen and the globus pallidus may result in a deterioration of the swallowing reflex, thereby increasing the risk of developing HCAP. In other words, the presence of the aforementioned cerebral infarction may serve as an indicator of underlying mechanisms that contribute to the development of pneumonia in dementia subtypes.

A dichotomy analysis demonstrated reproducibility in a previous report that patients with a blunted CRS (cough threshold concentration; 1.35 log mg/mL or higher) had a significantly higher incidence of pneumonia. Heretofore, the CRS has been reported to be affected by the female sex, lacunar infarctions in the basal ganglia, medication with dopaminergic agent, ACE inhibitor, neuroleptics, and gastroesophageal reflux (GER) [23]. However, these factors did not affect the incidence of HCAP in the study, although the existence of GER was investigated through interviews or medical records.

Additionally, neither xerostomia nor anti-cholinergic agent-induced hyposaliva was associated with the development of HCAP in the study. Consistent with the findings of a preceding prospective cohort study [5], the TOM in participants with advanced dementia was significantly lower than that observed in individuals in the MCI stage. As dementia progresses, salivary output may be compromised, which can lead to an increased risk of pneumonia due to proliferation of pathogenic bacteria.

### 4.2. The Number of Aspiration Pneumonia-Related Factors in HCAP by Dementia Subtype

Further, comprehensive investigation for AP-related factors including upper respiratory protective reflexes was reported. A LTSR more than 0.46 log sec (calculated to 2.91 s) and a CRS more than 1.35 log mg/mL are reasonable as cutoff values for developing HCAP, which correspond to previous references in which those values are 3.0 s and 1.35 log mg/mL, respectively [4].

Heretofore, deteriorated upper respiratory protective reflexes have been established as AP-related factors. However, as our study demonstrated, the participants in the PN group with both prolonged LTSR and blunted CRS were only approximately 40% (*p* = 0.009). The subsequent investigation of the remaining 60% of AP-related factors, excluding reflexes, extracted three factors of lacunar infarction, being of male sex, and smoking history.

Smoking is a prevalent custom among male individuals and has been identified as a potent risk factor for cerebrovascular disease, which is a primary cause of VaD [24]. Given that VaD accounted for 70% of all study subjects, bilateral lacunar infarctions were more prevalent among males (*p* = 0.033) (sub-analysis (%): hemi/bilateral: male; 37.8/62.2 vs. female; 55.0/45.0, respectively). Therefore, it might be taken for granted that these factors of developing HCAP in elderly individuals with dementia are extracted.

In summary, the study is the first report to identify AP-related factors, including upper respiratory protective reflexes, and to demonstrate a relationship between the possessed number of these factors and the incidence of HCAP.

### 4.3. Relationship Between Aspiration Pneumonia-Related Factors and Cognitive Function by Dementia Subtype

In our study, the LTSR prolonged as cognitive function declined in the advanced dementia stage. The volume of leukoaraiosis showed a significant correlation with cognition by MMSE (sub-analysis, *p* < 0.001), whereas lacunar infarction in the basal ganglia impacted the prolongation of the LTSR but not cognitive decline. In AD, the prolonged LTSR was reproducibly observed not in the MCI stage but in the severe dementia stage of AD [25]. In contrast, it was observed even in the MCI stage of VaD (*p* = 0.001). In other words, the clinical stages of dementia recognized the LTSR prolongation was different between AD and VaD.

An association between the impaired upper respiratory protective reflexes and cognitive dysfunction should be considered from the perspectives of the corticobulbar pathway and the putamen and globus pallidus in the basal ganglia. The corticobulbar pathway consists of a descending pathway from the motor cortex to the medulla oblongata and plays a crucial role in the transmission of swallowing motor functions [26]. On the other hand, the basal ganglia are involved in cognitive functions, motor planning, and execution where neurons regulate the activity of the cortico-brainstem pathway. In other words, the putamen/globus pallidus infarction may not only prolong the LTSR due to the delay of the axonal reflex caused by insufficient SP from the release of the dopaminergic nerves, but also indirectly weaken the corticobulbar pathway involved swallowing, whereas extensive degeneration of the cortico-brainstem pathway, such as severe advanced dementia, can synchronously show the deterioration of the upper respiratory protective reflexes, difficulty of swallowing motor functions, and severe cognitive dysfunction.

In summary, the presence of lacunar infarcts in the basal ganglia and different progressive patterns of overall brain atrophy suggest not only differences in the subtypes and clinical stages of dementia but also the underlying differences in the mechanisms of developing pneumonia.

The limitations of this study are as follows: AD participants were recruited on the basis of a definition that was provided in the text. They were confirmed to not have any visually identifiable cerebral vascular lesions on their brain MRI. Consequently, the sample size was constrained to 30 patients, yielding a post hoc power of 0.8 or less and a significant β error. Moreover, to ascertain whether a cohort of dementia patients manifesting five AP-related factors is more prone to future development of HCAP, a prospective cohort study is requisite.

In conclusion, the incidence of HCAP was higher in VaD than in AD. The upper respiratory protective reflexes of participants with AD or VaD deteriorated progressively with the progression of dementia. The deteriorated swallowing reflex in VaD participants, even at the MCI stage, may contribute to higher HCAP morbidity. Our study suggests that the presence of dementia and other AP-related factors, including deteriorated upper respiratory protective reflexes, lacunar infarctions in the bilateral basal ganglia, a history of smoking, and being of male sex, may predict the development of HCAP in elderly individuals with dementia.

## Figures and Tables

**Table 1:** Patient’s Characteristics.

	All Participants			AD Participants			VaD Participants		
	PN	CON	*p* Value	PN	CON	*p* Value	PN	CON	*p* Value
N	76	78	—	12	18	—	64	60	—
Sex (male n, (%))	47 (61.8)	27 (34.6)	0.001	6 (50.0)	5 (27.8)	0.266	41 (64.1)	22 (36.7)	0.002
Age mean ± SD (years old)	87.4 ± 5.3	88.3 ± 4.9	0.225	87.0 ± 5.1	86.6 ± 3.7	0.810	87.5 ± 5.4	88.8 ± 5.1	0.185
Smoking history n, (%)	30 (39.5)	18 (23.1)	0.028	7 (58.3)	3 (16.7)	0.045	23 (35.9)	15 (25.0)	0.187
Cognition									
MMSE score mean ± SD (score)	17.1 ± 8.8	15.2 ± 8.5	0.072	15.0 ± 8.2	14.1 ± 9.7	1.000	17.5 ± 8.9	15.6 ± 8.2	0.053
FAST medial value(quantile), (level)	6b (4–6e)	6c (4–6e)	0.829	6a (4–6c)	6c (4–7a)	0.632	6b (4–7a)	6b (4–6e)	0.580
Physical function									
PS score medial value(quantile), (level)	2 (1–3)	2 (1–3)	0.537	2 (2–3)	2 (1–3)	0.602	2 (1–3)	2 (1–3)	0.672
Barthel Index mean ± SD (score)	48.0 ± 36.3	46.3 ± 35.5	0.962	44.2 ± 42.1	51.7 ± 38.3	0.662	48.8 ± 35.5	44.8 ± 34.7	0.680
Eating ability									
FILS medial value(quantile), (level)	6 (2–8)	7 (5–10)	0.004	7 (5–7)	5 (4–9)	0.787	4 (2–8)	7 (5–10)	0.002
Comorbidities contributes to onset of AP									
Postoperative state of gastrointestinal tract, n (%)	7 (9.2)	2 (2.6)	0.096	2 (16.7)	1 (5.6)	0.548	5 (7.8)	1 (1.7)	0.209
COPD, n (%)	10 (13.2)	6 (7.7)	0.266	1 (8.3)	2 (11.1)	1.000	9 (14.1)	4 (6.7)	0.179
Medications which affect the development of pneumonia									
Neuroleptics, n (%)	4 (5.3)	1 (1.3)	0.207	1 (8.3)	0	0.400	3 (4.7)	1 (1.7)	0.620
Anticholinergic agent, n (%)	4 (5.2)	5 (6.4)	1.000	0	2 (11.1)	0.503	4 (6.3)	3 (5.0)	1.000
ACE-Inhibitor, n (%)	6 (7.9)	7 (9.0)	0.810	0	3 (16.7)	0.255	6 (9.4)	4 (6.7)	0.745
Dopamine-releasing hormone agent, n (%)	2 (2.6)	0	0.242	0	0	—	2 (3.1)	0	0.496
PDE-III Inhibitor, n (%)	4 (5.3)	4 (5.1)	1.000	1 (8.3)	3 (16.7)	0.632	3 (4.7)	1 (1.7)	0.620

AD; Alzheimer’s type dementia, VaD; vascular type of dementia, PN; participants with pneumonia, CON; participants without pneumonia, MMSE; Mini-Mental State Examination, FILS; Food Intake Level scale, FAST; Functional Assessment Staging, ACE; Angiotensin-Converting-Enzyme, PDE-III; Phosphodiesterase-III. Age, MMSE, FAST, Barthel Index, FILS: Mann-Whitney U test, sex, smoking history, comorbidities contribute to the development of aspiration pneumonia, participants who take medications that affect the development of pneumonia; chi-square test or Fischer’s probability test.

**Table 2:** Association of HCAP and aspiration pneumonia-related factor by dementia subtypes.

	All Participants					AD Participants			VaD Participants				
	PN	CON	*p* Value	Odds Ratio [95% CI]	*p* Value	PN	CON	*p* Value	PN	CON	*p* Value	Odds Ratio [95% CI]	*p* Value
LTSR mean ± SD, log sec.	0.52 ± 0.41	0.38 ± 0.33	0.010	3.98 [1.38–11.50]	0.011	0.37 ± 0.29	0.35 ± 0.40	0.573	0.55 ± 0.42	0.39 ± 0.31	0.008	4.71 [1.35–6.49]	0.015
CRS mean ± SD, log mg/mL	1.13 ± 0.49	1.02 ± 0.47	0.097	1.55 [0.74–3.25]	0.244	1.07 ± 0.45	0.98 ± 0.63	0.669	1.14 ± 0.50	1.03 ± 0.41	0.084	1.78 [0.75–4.24]	0.192
TOM mean ± SD, %	18.1 ± 9.6	16.6 ± 9.3	0.252	1.02 [0.98–1.06]	0.251	21.7 ± 9.5	15.1 ± 10.5	0.053	17.4 ± 9.5	17.1 ± 8.9	0.669	1.01 [0.97–1.05]	0.661

AD; Alzheimer’s type dementia, VaD; vascular type of dementia, PN; participants with pneumonia, CON; participants without pneumonia, LTSR; latency of the swallowing reflex, CRS; cough reflex sensitivity, TOM; tongue oral moisture. Dependent variable; presence or absence of pneumonia, Each individual variables; LTSR, CRS, and TOM, adjusted variables in all participants; sex, age, and MMSE, additional adjusted variable in VaD participants; presence or absence of lacunar infarction.

## Data Availability

The original contributions presented in this study are included in the article. Further inquiries can be directed to the corresponding author.

## References

[1] Teramoto S., Fukuchi Y., Sasaki H., Sato K., Sekizawa K., Matsuse T. (2008). High incidence of aspiration pneumonia in community- and hospital-acquired pneumonia in hospitalized patients: A multicenter, prospective study in Japan. J. Am. Geriatr. Soc..

[2] Mitchell S.L., Teno J.M., Kiely D.K., Shaffer M.L., Jones R.N., Prigerson H.G., Volicer L., Givens J.L., Hamel M.B. (2009). The clinical course of advanced dementia. N. Engl. J. Med..

[3] Data Base by The Ministry of Health, Labor and Welfare. https://www.mhlw.go.jp/content/001279920.pdf.

[4] Ebihara T. (2022). Comprehensive Approaches to aspiration pneumonia and dysphagia in the elderly on the disease time-axis. J. Clin. Med..

[5] Takeshita T., Tomioka M., Shimazaki Y., Matsuyama M., Koyano K., Matsuda K., Yamashita Y. (2010). Microfloral characterization of the tongue coating and associated risk for pneumonia-related health problems in institutionalized older adults. J. Am. Geriatr. Soc..

[6] Langmore S.E., Terpenning M.S., Schork A., Chen Y., Murray J.T., Lopatin D., Loesche W.J. (1998). Predictors of aspiration pneumonia: How important is dysphagia?. Dysphagia.

[7] Kobayashi S., Kubo H., Yanai M. (2007). Impairment of the swallowing reflex in exacerbations of COPD. Thorax.

[8] Marumo K., Homma S., Fukuchi Y. (1995). Post-gastrectomy aspiration pneumonia. Chest.

[9] Herzig S.J., LaSalvia M.T., Naidus E., Rothberg M.B., Zhou W., Gurwitz J.H., Marcantonio E.R. (2017). Antipsychotics and the Risk of Aspiration Pneumonia in Individuals Hospitalized for Nonpsychiatric Conditions: A Cohort Study. J. Am. Geriatr. Soc..

[10] Kose E., Hirai T., Seki T. (2018). Assessment of aspiration pneumonia using the Anticholinergic Risk Scale. Geriatr. Gerontol. Int..

[11] (2005). Guidelines for the management of adults with hospital-acquired, ventilator-associated, and healthcare-associated pneumonia. Am. J. Respir. Crit. Care Med..

[12] American Psychiatric Association (2003). Diagnostic and Statistical MANUAL of Mental Disorders.

[13] Hirata Y., Matsuda H., Nemoto K., Ohnishi T., Hirao K., Yamashita F., Asada T., Iwabuchi S., Samejima H. (2005). Voxel-based morphometry to discriminate early Alzheimer’s disease from controls. Neurosci. Lett..

[14] Minoshima S., Foster N.L., Kuhl D.E. (1994). Posterior cingulate cortex in Alzheimer’s disease. Lancet.

[15] Román G.C., Tatemichi T.K., Erkinjuntti T., Cummings J.L., Masdeu J.C., Garcia J.H., Amaducci L., Orgogozo J.M., Brun A., Moody D.M. (1993). Vascular dementia: Diagnostic criteria for research studies. Report of the NINDS-AIREN International Workshop. Neurology.

[16] Folstein M.F., Folstein S.E., McHugh P.R. (1975). “Mini-mental state”. A practical method for grading the cognitive state of patients for the clinician. J. Psychiatr. Res..

[17] Reisberg B. (1988). Functional assessment staging (FAST). Psychopharmacol. Bull..

[18] Mahoney F.I., Barthel D.W. (1965). Functional Evaluation: The Barthel Index. Md. State Med. J..

[19] Kunieda K., Ohno T., Fujishima I., Hojo K., Morita T. (2013). Reliability and validity of a tool to measure the severity of dysphagia: The Food Intake LEVEL Scale. J. Pain Symptom Manag..

[20] Gonzalez-Fernandez M., Kleinman J.T., Ky P.K., Palmer J.B., Hillis A.E. (2008). Supratentorial regions of acute ischemia associated with clinically important swallowing disorders: A pilot study. Stroke.

[21] Steinhagen V., Grossmann A., Benecke R., Walter U. (2009). Swallowing disturbance pattern relates to brain lesion location in acute stroke patients. Stroke.

[22] Lowell S.Y., Poletto C.J., Knorr-Chung B.R., Reynolds R.C., Simonyan K., Ludlow C.L. (2008). Sensory stimulation activates both motor and sensory components of the swallowing system. Neuroimage.

[23] Ebihara S., Ebihara T., Yamasaki M., Asada M., Yamanda S., Niu K., Sasaki H., Arai H. (2007). Contribution of gastric acid in elderly nursing home patients with cough reflex hypersensitivity. J. Am. Geriatr. Soc..

[24] Podcasy J.L., Epperson C.N. (2016). Considering sex and gender in Alzheimer disease and other dementias. Dialogues Clin. Neurosci..

[25] Wada H., Nakajoh K., Satoh-Nakagawa T., Suzuki T., Ohrui T., Arai H., Sasaki H. (2001). Risk factors of aspiration pneumonia in Alzheimer’s disease patients. Gerontology.

[26] DeMyer W. (1998). Neuroanatomy.

